# Morphogenetic Variability as Potential Biomarker of Functional Outcome After Ischemic Stroke

**DOI:** 10.3390/brainsci9060138

**Published:** 2019-06-14

**Authors:** Milan Savic, Suzana Cvjeticanin, Milica Lazovic, Ljubica Nikcevic, Ivana Petronic, Dragana Cirovic, Dejan Nikolic

**Affiliations:** 1 Special Hospital for Cerebrovascular Disorders “Sveti Sava”, 11000 Belgrade, Serbia; milan.savic@svetisava.rs (M.S.); ljubicanikcevic@yahoo.com (L.N.); 2 Institute for Human Genetics, Faculty of Medicine, University of Belgrade, 11000 Belgrade, Serbia; cujasimsi@gmail.com; 3 Faculty of Medicine, University of Belgrade, 11000 Belgrade, Serbia; lazovicmilica15@gmail.com (M.L.); ivana.pm@live.com (I.P.); cirovicdragana@yahoo.com (D.C.); 4 Institute for Rehabilitation, 11000 Belgrade, Serbia; 5 Physical Medicine and Rehabilitation Department, University Children’s Hospital, 11000 Belgrade, Serbia

**Keywords:** stroke, homozygous recessive characteristics, functional outcome, age

## Abstract

The aim of our study was to evaluate the role of morphogenetic variability in functional outcome of patients with ischemic stroke. The prospective study included 140 patients with acute ischemic stroke, all of whom were tested upon: admission; discharge; one month post-discharge; and three months post-discharge. The age was analyzed, as well. The Functional Independence Measure (FIM) test and the Barthel Index (BI) were used for the evaluation of functional outcomes for the eligible participants. We analyzed the presence of 19 homozygous recessive characteristics (HRC) in the studied individuals. There was a significant change in FIM values at discharge (*p* = 0.033) and in BI values upon admission (*p* = 0.012) with regards to the presence of different HRCs. Age significantly negatively correlated for the FIM score and BI values at discharge for the group with 5 HRCs (*p* < 0.05), while for BI only, negative significant correlation was noticed for the group with 5 HRCs at three months post-discharge (*p* < 0.05), and for the group with 3 HRCs at one month post-discharge (*p* < 0.05) and three months post-discharge (*p* < 0.05). Morphogenetic variability might be one among potentially numerous factors that could have an impact on the response to defined treatment protocols for neurologically-impaired individuals who suffered an ischemic stroke.

## 1. Introduction

Most stroke patients demonstrate functional improvements over time. Such improvements might be connected with compensatory processes, that could be to the certain degree explained by the brain spasticity [1]. Previous studies have evaluated the role of candidate genes on neurological deficit, functional ability, and social participation in stroke patients [2,3,4]. Further, it has been suggested that genetic variations might be used in the prediction of neural injury recovery [5]. 

It should be noticed that the rehabilitation outcome of patients who suffer from neurological conditions rests on a complex interaction among the patient’s baseline status, modifiable and non-modifiable individual factors, and the treatment program [6]. However, the presence of different variations of functional outcomes after a stroke is still not fully elucidated. This might be explained by the fact that different parts of the brain, along with different degrees of neurological injury and present comorbidities, influence an individual’s functional recovery potential. Therefore, functional outcome prediction is complex and difficult. Thus, factors specific to pathophysiological stroke subtypes, as well as biological and genetic factors, should be evaluated [2]. Furthermore, a better understanding of biomarkers in motor recovery after the stroke would be of great value for creating personalized rehabilitation treatment modes and proper selection for trials dealing with rehabilitation interventions [7].

The aim of our study was to evaluate the role of morphogenetic variability in the functional outcome of patients with ischemic stroke.

## 2. Methods

### 2.1. Study Group

The prospective study included 140 patients who were diagnosed and treated with acute ischemic stroke by a board-certified neurologist and referred to rehabilitation treatment that was conducted by board-certified physiatrist at a specialty hospital for cerebrovascular diseases, “Sveti Sava” in Belgrade. The participants were tested on four different occasions (at admission—Group 1; upon discharge—Group 2; one month post-discharge—Group 3; and three months post-discharge—Group 4). The age was analyzed, as well (participants were between 65–80 years old). Enrolled patients were included in a standard physiotherapy program after the stabilization of overall health parameters. The physiotherapy included kinesiotherapy procedures five times per week. All eligible participants and/or legal guardians were informed about the study protocol and consent was obtained. The study was approved by Institutional Review Board and followed the principles of good clinical practice.

### 2.2. Functional Status Estimation

The Functional Independence Measure (FIM) test was performed for the evaluation of functional (motor and cognitive) status of the eligible participants. The FIM is composed of 18 items in total, where 13 refer to the motor subscale and five are cognitive subscales [8]. The motor subscale gives the information regarding: self-care, sphincter control, transfers, and locomotion, while the cognitive subscale analyzes communication and social cognition. There is a seven-grade scoring system for every item, where the total sum can range between 18–126 [8]. 

The Barthel Index (BI) was used to measure the functional outcome in the tested patients. The scale is composed of 10 tasks and was scored from 0 to 100, where lower scores represent greater nursing dependency [9].

### 2.3. Tested Homozygous Recessive Characteristics

We implemented the homozygous recessive characteristics (HRC) test [10,11,12] to estimate the degree of recessive homozygosity in the eligible participants. The HRC test was developed for the evaluation of the proportion of HRCs that are clearly expressed and considered as qualitative traits, thus being markers of chromosomal homozygosities in every individual [12,13,14,15,16,17,18]. The studied HRCs are the markers of genes located on different chromosomes [19]. We analyzed the presence of 19 HRCs in the studied individuals, only marking as the present trait characteristics that appeared extreme. In the region of the human head, we tested 13 HRCs: attached ear lobe (OMIM number 128900), continuous frontal hair line (OMIM number 194000), blue eyes (gene location 15q12, 15q13, OMIM number 227220; 5p13 OMIM number 227240; 14q32.1, OMIM number 210750; 9q23 OMIM number 612271), straight hair (1q21.3, OMIM number 139450), soft hair and blond hair (gene location 15q12, 15q13, OMIM number 227220; 14q32.1, OMIM number 210750; 12q21.3 OMIM number 611664; 11q13.3, OMIM number 612267), double hair whorl, opposite hair whorl orientation (OMIM number 139400), an inability to roll, fold, and curve the tongue (OMIM number 189300), ear without Darwinian notch, ability to produce a guttural “r”, and color blindness (gene location Xq28, OMIM number 303800). In human arms, we tested six HRCs: proximal thumb hyperextensibility, index finger longer than the ring finger (OMIM number 136100), left-handedness (gene location 2p12-q22, OMIM number 139900), right thumb over left thumb (hand clasping) (OMIM number 139800), top joint of the thumb >45°, and three tendons in the wrist (OMIM) [20].

### 2.4. Statistical Analysis

The results were presented as mean values (MV) with standard deviation (SD). A one-way ANOVA test was performed to evaluate the presence of statistical significance between continuous variables. The Mann–Whitney U test was done to analyze the statistical significance in functional scores changes between two different times of observation. To estimate the degree of correlation between age and scores of functional tests that were performed in defined times of observation, we used Spearman’s correlation test. For evaluation and quantification of variability that can be explained between different functional scores in defined time of observation among individuals with different amount of HRCs, we introduced η^2^ = Sum of squares (Between groups)/Sum of squares (Total) × 100, where sum of squares was gained from the one-way ANOVA test, and the results were presented as percentage (%) [21]. Statistical significance was set at *p* < 0.05.

## 3. Results

**Figure 1:**
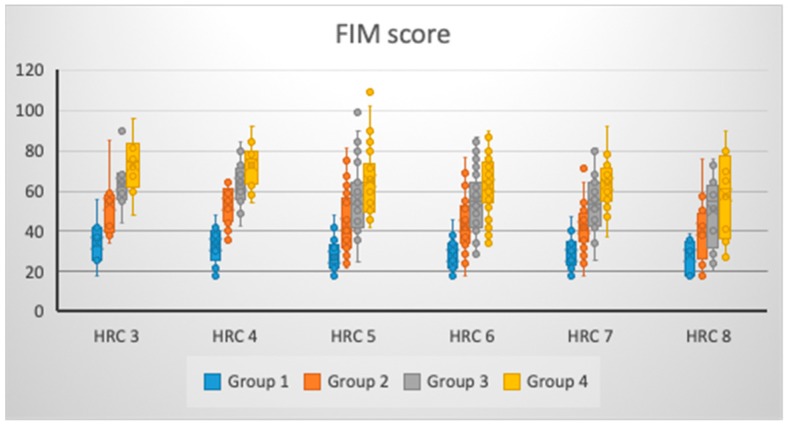
FIM score changes at different times of observation, given the number of HRCs.

There was no significant change in FIM values for Group 1 (*p* = 0.077), Group 3 (*p* = 0.141) and Group 4 (*p* = 0.075) with regards to the presence of different amount of HRCs, while a significant change was noticed for Group 2 (*p* = 0.033) (Table 1 and Figure 1). However, as the number of HRCs increased, there was a decrease in FIM score in Group 2 and Group 3; however, in Group 1 and Group 4, patients with three and four HRCs had higher FIM scores versus those with 5–8 HRCs, pointing to the unchanged trend that an increased level of genetic homozygosity leads to a less favorable FIM score. For all groups (η^2^_Group1_ = 7.08%; η^2^_Group2_ = 8.55%; η^2^_Group3_ = 5.94%; and η^2^_Group4_ = 7.12%), the low effects size of a different amount of HRCs was noticed to be associated with FIM scores (Table 1).

**Figure 2:**
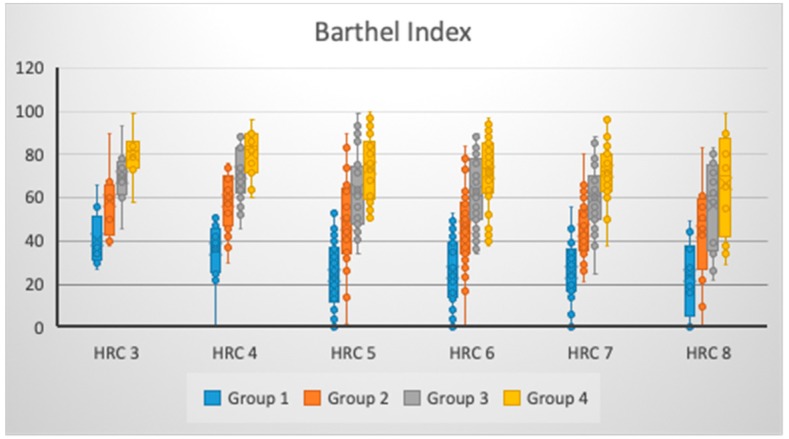
BI changes at different times of observation, given the number of HRCs.

We have shown that there was non-significant change in the BI values for Group 2 (*p* = 0.074), Group 3 (*p* = 0.117), and Group 4 (*p* = 0.159) regarding the presence of different amounts of HRCs, while significant change was noticed for Group 1 (*p* = 0.012) (Table 1 and Figure 2). However, as the number of HRCs increased, there was a decrease in BI values in Group 2; however, in Groups 1, 3, and 4, patients with three and four HRCs had higher BI scores versus those with 5–8 HRCs, pointing to the unchanged trend that an increased level of genetic homozygosity leads to a less favorable BI score. For all groups (η^2^_Group1_ = 10.27%; η^2^_Group2_ = 7.13%; η^2^_Group3_ = 6.30%; and η^2^_Group4_ = 5.70%), the low effects size of a different amount of HRCs was noticed to be associated with BI scores, with the highest effects size for Group 1 (Table 1).

In Table 2, we presented a statistical interpretation of changes in functional scores for both FIM and BI between defined times of observation. There was significant change in the functional scores between admission and discharge, between admission and one month post-discharge, and between one and three months post-discharge, for both FIM and BI (Table 2). A significant difference in the functional scores for both FIM and BI between discharge and one month post-discharge, between discharge and three months post-discharge, and between one and three months post-discharge was noticed for individuals with a number of HRCs between 4–7 (Table 2). For the BI, a significant change in scores was noticed, as well, between discharge and three months post-discharge for groups of individuals with the number of HRCs of 3 and 8 (Table 2).

Age significantly negatively correlated for FIM score values at discharge for the group of tested individuals with 5 HRCs (*r* = −0.418; *p* < 0.05), while for BI, negative significant correlation was noticed for tested individuals with 5 HRCs at discharge (*r* = −0.371; *p* < 0.05) and three months post-discharge (*r* = −0.362; *p* < 0.05), and for tested individuals with 3 HRCs one month post-discharge (*r* = −0.756; *p* < 0.05) and three months post-discharge (*r* = −0.756; *p* < 0.05) (Table 3). 

## 4. Discussion

Despite the fact that numerous genetic loci were found to be associated with stroke risk, the role of genes on functional outcome in stroke patients is less clear [22]. This is of particular importance since 28% of stroke survivors are still dependent on others after one year after the event [22]. Stroke recovery biomarkers were proposed in the study of Boyd at el. for the purpose of better understanding and ability to predict long-term outcomes after a stroke [23]. Therefore, we proposed the evaluation of morphogenetic variability on the functional outcome in patients with ischemic stroke as an additional tool that would bring better understanding of the possible underlining mechanisms responsible for better outcome in these patients.

Our findings demonstrated that, as the number of tested HRCs increased in studied individuals, there was decrease in the values of FIM and BI scores on all tested occasions. However, only significant changes of these scores was noticed for FIM after discharge, while for BI this occurred at admission. Presence of such differences might be explained by the fact that different determinants were tested in FIM and BI scores. When graphically presented, it was noticed that different variations in FIM and BI scores at defined time points were present for patients with a different amount of tested HRCs. This might bring to light the assumption that both genetic and individual (modifiable) factors could have, to a certain degree, the potential to influence functional outcomes after a stroke. Moreover, we might stress that the possible presence of significant population-genetic differences between stroke patients with various amounts of tested HRCs could exist, with certain preferential phenotypes for greater potential of better functional outcome. A higher degree of genetic homozygosity, followed by a decrease in functional improvement and increase in variability of functional outcome, particularly three months post-discharge for both FIM and BI, might bring these patients into a specific state of genetic-physiological homeostasis where certain mechanisms will influence the potential for functional recovery, along with medicamentous and rehabilitation treatment. Furthermore, it is worth mentioning that an increase in genetic (recessive) homozygosity could enlarge the genetic load degree, potentially causing a decrease in body immunity [24], with the potential effects on the ability of the organism to respond in a less wide variation to the treatment methods, thus reducing the potential for functional improvement.

Numerous studies we performed show that a higher degree of genetic homozygosity leads to a decrease in genetic variability in what may change, together with an increase of genetic loads and genetic physiological homeostasis [12,13,17,24,25,26]. We may presume that all this influence decreases the number of possible body responses to action in numerous environmental factors. Taking this into the consideration, we may explain the less favorable results on the rehabilitation tests of those individuals with higher degree of genetic homozygosity compared to patients with lower level of homozygosity.

In our study, when age was introduced as a correlation variable, we noticed that significant correlations were different for FIM versus for BI. It should be stressed that there are still opposing opinions regarding the sensitivity of FIM and BI scores in evaluation of stroke patients. Dromerick et al. [27] stated that FIM is more sensitive, while Sangha et al. [28] stated that BI was cited in trials of superior quality. Our study pointed out that BI was somehow more sensitive versus the FIM score when age was correlated with regard to the different number of HRCs. This might suggest that certain phenotypes with regard to age could have greater potential for different aspects of functional outcomes in ischemic stroke patients. Therefore, in a different age group of stroke onset, individuals with a different amount of tested HRCs might be better candidates for defined treatment modes in order to achieve optimal functional improvement of certain functional aspects. Our findings imply the justification for the proposal of personalized rehabilitation treatment modes in patients with ischemic stroke. 

There are several limitations regarding this study. The first limitation refers to the study group, where patients from one population (Serbian)were studied. Another limitation to be considered is potential specific variations, modifiable and non-modifiable, in different populations, and phenotype classes. Furthermore, the number of patients should be considered as limiting factor; therefore, a larger sample study is advised.

## 5. Conclusions

In conclusion, our findings will contribute to the better understanding of the potential determinants which could predict response and, finally, outcome to defined treatment protocols, particularly in neurological patients with various degrees of functional disabilities, thus providing important information to practitioners in clinical settings for establishing optimal clinical decision-making strategies. By better understanding of selective biomarkers and their role in stroke recovery, we could be less challenged in the dilemma of the patient’s potential for recovery. Morphogenetic variability, therefore, might be one among potential numerous factors that could have impact on response to a defined treatment protocols for neurologically-impaired individuals who suffered the ischemic stroke. 

## Figures and Tables

**Table 1:** Functional scores at different times of observation, given the number of homozygous recessive characteristics (HRCs). FIM = Functional Independence Measure; BI = Barthel Index; MV = mean values; SD = standard deviation.

HRC	Group 1 (MV ± SD) 95% CI	Group 2 (MV ± SD) 95% CI	Group 3 (MV ± SD) 95% CI	Group 4 (MV ± SD) 95% CI
	FIM Score			
3 (*N* = 8)	34.38 ± 12.19 24.19–44.56	53.13 ± 15.87 39.86–66.39	63.63 ± 13.30 52.51–74.74	73.00 ± 14.90 60.54–85.46
4 (*N* = 17)	33.12 ± 9.11 28.43–37.80	53.35 ± 10.02 48.20–58.51	63.24 ± 10.87 57.65–68.82	72.47 ± 10.89 66.87–78.07
5 (*N* = 30)	27.20 ± 8.21 24.13–30.27	43.27 ± 15.42 37.51–49.02	55.93 ± 17.92 49.24–62.62	65.30 ± 16.73 59.05–71.55
6 (*N* = 43)	28.02 ± 7.45 25.73–30.32	42.91 ± 13.81 38.66–47.16	54.74 ± 15.36 50.02–59.47	62.95 ± 14.74 58.42–67.49
7 (*N* = 30)	27.97 ± 7.78 25.06–30.87	42.17 ± 11.64 37.82–46.51	53.73 ± 13.41 48.73–58.74	63.33 ± 12.07 58.83–67.84
8 (*N* = 12)	28.17 ± 8.03 23.06–33.27	41.42 ± 16.51 30.93–51.91	50.25 ± 18.38 38.57–61.93	58.08 ± 21.51 44.42–71.75
*p* *	0.077	0.033	0.141	0.075
η^2^ (%)	7.08%	8.55%	5.94%	7.12%
**HRC**	**BI**			
3 (*N* = 8)	40.63 ± 13.42 29.41–51.84	58.38 ± 16.54 44.54–72.21	69.50 ± 13.61 58.12–80.88	79.88 ± 11.93 69.90–89.85
4 (*N* = 17)	36.29 ± 13.37 29.42–43.17	58.47 ± 13.78 51.39–65.56	71.59 ± 13.36 64.72–78.46	80.29 ± 11.04 74.62–85.97
5 (*N* = 30)	24.17 ± 16.08 18.16–30.17	48.20 ± 20.24 40.64–55.76	63.43 ± 17.80 56.79–70.08	74.10 ± 15.14 68.45–79.75
6 (*N* = 43)	25.47 ± 14.79 20.91–30.02	46.49 ± 17.23 41.18–51.79	62.65 ± 15.69 57.82–67.48	71.58 ± 16.13 66.62–76.55
7 (*N* = 30)	25.23 ± 14.69 19.75–30.72	45.33 ± 13.70 40.22–50.45	60.10 ± 14.62 54.64–65.56	72.07 ± 13.25 67.12–77.01
8 (*N* = 12)	24.17 ± 17.37 13.13–35.20	44.58 ± 23.40 29.72–59.45	56.58 ± 21.24 43.09–70.08	66.58 ± 23.35 51.75–81.42
*p* *	0.012	0.074	0.117	0.159
η^2^ (%)	10.27%	7.13%	6.30%	5.70%

HRC, homozygous recessive characteristics; FIM, Functional Independence Measure; BI, Barthel Index; MV, mean value; SD, standard deviation; CI, confidence interval; *, one-way ANOVA.

**Table 2:** Statistical interpretation of functional score changes between defined times of observation.

HRC	Group 1/2 *	Group 1/3 *	Group 1/4 *	Group 2/3 *	Group 2/4 *	Group 3/4 *
	FIM Score					
3	0.027	0.002	0.001	0.073	0.032	0.171
4	<0.000	<0.000	<0.000	0.025	<0.000	0.029
5	<0.000	<0.000	<0.000	0.004	<0.000	0.023
6	<0.000	<0.000	<0.000	0.001	<0.000	0.018
7	<0.000	<0.000	<0.000	0.001	<0.000	0.006
8	0.014	0.006	0.002	0.126	0.064	0.298
**HRC**	**BI**					
3	0.024	0.003	0.001	0.093	0.032	0.103
4	<0.000	<0.000	<0.000	0.023	<0.000	0.048
5	<0.000	<0.000	<0.000	0.007	<0.000	0.020
6	<0.000	<0.000	<0.000	<0.000	<0.000	0.008
7	<0.000	<0.000	<0.000	<0.000	<0.000	0.003
8	0.016	0.002	<0.000	0.194	0.040	0.285

HRC, homozygous recessive characteristics; FIM, Functional Independence Measure; BI, Barthel Index; *, Mann–Whitney U test.

**Table 3:** Correlation between age and functional scores in defined time of observation.

HRC	Group 1/Age *	Group 2/Age *	Group 3/Age *	Group 4/Age *
	FIM Score			
3	0.205	−0.036	0.167	0.357
4	0.127	−0.005	−0.088	0.041
5	−0.271	−0.418 **	−0.316	−0.318
6	0.000	−0.238	−0.122	−0.112
7	−0.108	−0.137	−0.037	0.050
8	0.391	0.205	−0.172	−0.158
**HRC**	**BI**			
3	0.548	−0.036	−0.756 **	−0.756 **
4	0.019	0.071	−0.079	0.018
5	−0.255	−0.371 **	−0.329	−0.362 **
6	−0.261	−0.211	−0.102	−0.035
7	−0.177	−0.001	−0.020	0.126
8	0.000	0.320	−0.160	−0.174

HRC, homozygous recessive characteristics; FIM, Functional Independence Measure; BI, Barthel Index; *, Spearman’s Correlation; **, *p* < 0.05.
